# Diagnosing Oncocytoma by Core Needle Biopsy: A Single-Center Experience

**DOI:** 10.1155/2022/1589040

**Published:** 2022-08-29

**Authors:** Chen Mayer, Yasmin Abu-Ghanem, Zohar A. Dotan, Iris Barshack, Eddie Fridman

**Affiliations:** ^1^Institute of Pathology, Sheba Medical Center, Tel Hashomer, Affiliated with Tel Aviv University, Tel Aviv, Israel; ^2^Urology Department, Sheba Medical Center, Tel Hashomer, Affiliated with Tel Aviv University, Tel Aviv, Israel; ^3^Faculty of Medicine, Tel Aviv University, Tel Aviv, Israel

## Abstract

**Background:**

Oncocytoma is one of the most common benign kidney tumors, accounting for 3–7% of all solid renal masses. Diagnosing oncocytomas using renal biopsy remains a controversy in the uro-pathologic community. With the increasing use of biopsies for assessment of renal lesions, reaching this pathologically benign diagnosis may prevent further surgical measures and have significant clinical benefit.

**Objective:**

To demonstrate our center's results using renal biopsy to diagnose oncocytomas and to suggest that this diagnosis can be made with high success rates.

**Design:**

, *Setting*, *and Participants*. From our center's database, we retrospectively identified and retrieved all cases of oncocytoma diagnosed between the years 2011 and 2020 by renal biopsy. Medical records of those patients were then reviewed to view follow-up meetings and imaging of the lesions biopsied. *Outcome Measurements and Statistical Analysis*. In 21 biopsies performed on 19 patients, diagnosis was supported by subsequent follow-up averaging at 3.44 years per patient. *Results and Limitations*. The lesions exhibited benign behavior during follow-up after biopsy, consistent with the diagnosis of oncocytoma.

**Conclusions:**

Our study demonstrates that with good patient selection and proficient cooperation between urologists, radiologists and dedicated uro-pathologists, correctly diagnosing oncocytomas using RCB is a viable task. *Patient Summary*. Oncocytomas are benign lesions of the kidney. In our study, we reviewed all cases of oncocytomas pathologically diagnosed using renal biopsy from our center's database. We found that in subsequent follow-up later to biopsy, the lesions displayed benign behavior consistent with oncocytoma. The use of percutaneous biopsies to reach this diagnosis could save patients more extensive surgeries.

## 1. Introduction

Renal oncocytoma is one of the most common benign kidney tumors, accounting for 3–7% of all solid renal masses [1, 2] and up to 18% of small renal masses measuring less than 4 cm [3].

Management of solid renal masses has until recently involved surgical excision for both diagnostic and therapeutic purposes; however, up to a third of asymptomatic radiologically suspicious renal masses measuring less than 5 cm are proven to be benign lesions on final pathological examination following specimen excision [4–8].

With increased popularity and improvements to imaging modalities, up to 70% of renal cortical tumors are now discovered incidentally, most with a median tumor size of less than 4 cm [9, 10]. Radiological characteristics suggestive of oncocytoma include sharp demarcation and a sharp central stellate scar, only seen in a third of cases. Despite improvements, it is still difficult to reliably distinguish between benign and malignant tumors, radiologically and clinically, without pathologic diagnosis [11]. In many centers, renal percutaneous core biopsies (RCBs) of kidney masses are now increasingly performed to establish pathologic diagnosis, in order to guide following therapy [12–15]. In earlier studies, the diagnostic accuracy of the core needle biopsy was somewhat unsatisfactory, with specific regard given to the misdiagnosis of oncocytic lesions [4, 16].

Diagnosing renal oncocytic neoplasms on biopsy remains challenging due to the considerable morphologic overlap among oncocytoma and its malignant counterparts which may mimic histological appearance, such as chromophobe, clear cell carcinoma, and others. Morphologic features and in selected cases immunohistochemistry are often helpful in this distinction [17], but there is significant controversy regarding the diagnosis of oncocytoma on biopsy alone.

In this study, we shall exhibit our center's experience with diagnosing oncocytomas on renal biopsies during the last 10 years and emphasize the value of pathologist's experience and patient selection in tackling these diagnoses.

**Figure 1:**
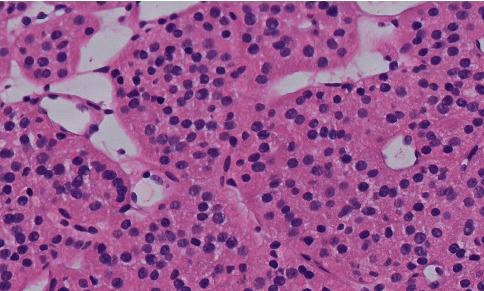
Oncocytoma biopsy specimen, H&E staining, ×20 magnification, digitally scanned.

Histopathological examination using hematoxylin and eosin (H&E) staining is the first and most important step in the diagnostic approach to epithelial renal neoplasms. Renal oncocytomas are characterized by “oncocytes,” which are by definition large neoplastic cells with intensely eosinophilic granular cytoplasm that results from large number of mitochondria (see Figure 1). Oncocytomas are found in a number of organs and have been described in the thyroid, salivary, parathyroid, and adrenal glands and other anatomical sites [18]. The cell type preceding development of oncocytomas is unknown, with most pathologists suggesting a distal tubular origin for renal oncocytomas [19, 20].

Renal oncocytoma has a variable morphological appearance. Cells are usually arranged in solid compact nests (acinar growth exemplar) and/or in cords, tubules, and sheets of trabeculae (tubulo-cystic arrangement) that are separated by loose edematous fibrous of hyalinized stroma. However, papillary and cystic architecture may occur, which are composed of regularly large oncocytes or small basophilic cells. The differentiation of two cell types has been discussed [21]. The predominant classic form of cells, the “oncocytes,” corresponds as mentioned above to round-to-polygonal cells with densely granular eosinophilic cytoplasm, round and uniform nuclei with finely distributed chromatin, and a centrally placed prominent nucleolus. A smaller population of cells named “oncoblasts” has less conspicuous, paler, scanty, and granular cytoplasm, a high nuclear/cytoplasmic ratio, and dense dark hyperchromatic nuclei. Bizarre, polyploidy, and enlarged nuclei, which are characteristic for endocrine adenomas, might be scattered throughout the lesion, but mitoses are absent.

The subset of renal oncocytic lesions includes three tumor types that exist on a clinic-pathologic continuum, ranging from benign renal oncocytoma discussed in this article, to indolent hybrid oncocytic/chromophobe renal tumor (HOCT) to the malignant chromophobe renal cell carcinoma [22]. By conventional pathologic examination, HOCTs harbor a mixture of cells with morphologic and immunophenotypic features that overlap with those of renal oncocytoma and chromophobe renal cell carcinoma. To note, the aforementioned hybrid tumors arise in several distinct settings: as part of multiple tumors in cases of renal oncocytosis, as tumors arising in patients with Birt–Hogg–Dube syndrome, and as sporadic tumors. Consequently, pathologists are reluctant to diagnose renal oncocytoma, particularly on the basis of core needle biopsy sampling, resulting in the non-specific and suboptimal biopsy diagnosis of “renal oncocytic neoplasm,” which is rendered in up to 11% of biopsied renal masses, in some studies [23]. New entities described on the oncocytic spectrum include low-grade and high-grade oncocytic tumors, described in articles from recent years [24].

The diagnosis of renal oncocytoma can be relatively simple in experienced hands, though difficulties may arise when this neoplasm shows atypical morphology or is part of a metastasizing disease, such seen in metastasizing oncocytoma —a rare scenario [25, 26]. Additional studies such as electron microscopy, chromosomal analysis, and immunohistochemistry might help in achieving a correct diagnosis in difficult cases [27–30].

## 2. Materials and Methods

The study was approved by the Helsinki Ethical Committee.

We retrospectively identified and retrieved from the Chaim Sheba hospital pathologic database all cases of oncocytoma diagnosed between the years 2011 and 2020, amounting at 79 cases of oncocytomas diagnosed pathologically. These cases included all specimens, from resected specimens to biopsies taken from kidney lesions. From these cases, we extracted cases first diagnosed using percutaneous biopsy alone, resulting in 21 biopsies taken from a total of 19 patients. The medical records of those patients were reviewed to obtain demographics, clinical history, imaging results, treatments received, and follow-up information. No cases were excluded other than considerations mentioned above.

To note, no lesions were reported as malignant on biopsy and later reported as an oncocytoma on the resected specimen.

Biopsies were performed by interventional radiologists under computed tomography guidance with an 18-gauge needle. All biopsy specimens were then fixed in 10% formalin and underwent routine laboratory processing.

**Figure 2:**
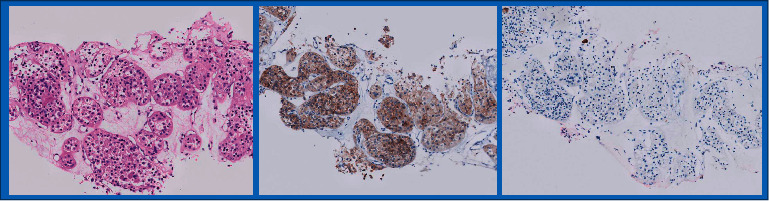
Typical immunostaining of oncocytomas, as seen in one of the cases detailed (left to right: H&E staining, positive C-KIT staining, and negative CK7 staining).

Histological diagnoses on all specimens were obtained from original pathology reports, most of them signed by a single dedicated uro-pathologist. Immunohistochemical stains were performed on 16 of the 21 biopsies reviewed, based on amount of tissue received and diagnostic necessity. Among stains used were C-KIT, CK7, CD10, CA IX, and Vimentin, according to current consensus (see Figure 2).

## 3. Results

Biopsies were performed during the years 2012–2020, with a mean age of 67 (ranged 8–92). In the patient group, there were 4 women and 15 men, creating a 1 : 3.75 ratio. Average follow-up time on patients was approximately 3.77 years following biopsy (ranged 0–10.38 years). Most biopsy cases (57%) had a follow-up time of over three years. To note, 2 patients were lost to follow-up (see Table 1).

Average size of lesion biopsied was approximately 3 cm (ranged 0.5–9.5 cm). Eight (38%) and thirteen (62%) of the lesions were located in the left kidney and right kidney, respectively. Four (21%) of the patients were diagnosed with multiple lesions in the same kidney, and six (29%) patients diagnosed with bilateral oncocytomas.

Only two patients underwent a nephrectomy following biopsy—one due to growth of the lesion by 35% during a three-year follow-up and the other due to patient preference. Both lesions were confirmed microscopically after nephrectomies as oncocytomas. None of the other lesions grew by more than 0.5 cm per year, as detailed in current follow-up guidelines.

In 16 of 21 biopsies reviewed, immunohistochemistry was performed to establish the diagnosis of oncocytoma, while in remaining cases, none was needed for diagnosis.

No surgical complications during or following biopsy procedure were observed.

## 4. Discussion

Until recently, renal biopsy was thought to be inaccurate in the aforementioned scenario and potentially dangerous. Technological developments and proper patient selection using advanced radiologic techniques have changed diagnostic approach, bringing with it new challenges to uro-pathologists in large medical centers.

Studies have shown conflicting results regarding the ability of correctly diagnosing oncocytomas in RCBs, with some studies advising primary total resection of lesions suspected as oncocytomas without biopsy.

Diagnosing this benign lesion based on biopsy alone prevents these patients from undergoing unnecessary nephrectomies, even if only performed partially. Even to this day, these operations hold significant surgical and post-operational risks. Of course, misdiagnosing these lesions as benign when they are in fact malignant could cause catastrophic outcomes for the patient on follow-up.

In our study, we exhibited all 21 biopsies signed out as oncocytomas in Sheba Medical Center between the years 2012 and 2020 with a follow-up period averaging at 3.77 years. The diagnosis was supported by subsequent long follow-up showing benign behavior, consistent with the diagnosis of oncocytoma. In the time period mentioned, there were no cases whose diagnosis was later changed by clinical events. As stated above, most biopsies were examined by a specialized uro-pathologist. Immunohistochemistry was helpful in most cases for establishing histological diagnosis.

A limitation of this study is the inability to definitively prove the diagnosis done by biopsy, by comparing it to that of the resected lesion. Follow-up time used to establish our diagnoses was over 3 years on average, which in some lesions may not be enough to assert the benign diagnosis. For example, rare cases of renal cell carcinoma and chromophobe carcinoma may grow slowly or not grow at all in the course of that time.

Our study demonstrates that with good patient selection and proficient cooperation between urologists, radiologists and dedicated uro-pathologists, correctly diagnosing oncocytomas using RCB is a viable task.

## Figures and Tables

**Table 1:** Clinical and pathologic data.

^#^			Clinical data				Pathologic data	
Patient^#^	Age	Gender	Single/multiple lesion	Bilateral	Sonographic size (cm)	Follow-up (yrs)	Side of Bx	Immuno-stains
1	76	m	Multiple	Bilateral	2.5	0.82	R	None performed
2, 1	69	m	Multiple	Bilateral	2.8	4.57	L	Positive for CD10, C-KIT, and CK7
2, 2	69	m	Multiple	Bilateral	1.3	4.57	R	Positive for CD10, C-KIT, and CK7
3	70	m	Single	One-sided	3	5.43	R	Positive for CD10, p504s, and C-KIT
4	35	f	Single	One-sided	2.8	3.59	L	Negative for colloidal iron, CK7, and C-KIT
5	69	m	Single	One-sided	2.5	5.18	L	Positive for C-KIT and CK7
6	65	m	Single	One-sided	9.5	5.21	R	Positive for CK7, C-KIT, CD10, and p504s
7	74	m	Single	One-sided	3	0.85	L	Positive for C-KIT, CD10, CK7, and p504s
8	8	m	Single	One-sided	0.5	6.89	R	Positive for MNF116, p504s, and CD10
9	79	f	Single	Bilateral	2.5	3.90	L	Positive for PAX8, C-KIT, and CK7
10	52	m	Single	One-sided	2.8	2.90	R	Positive for MNF116, CK7, and C-KIT
11	81	m	Single	Bilateral	4	4.19	R	None performed
12	83	m	Multiple	Bilateral	2.5	0.00	R	Positive for MNF116, CD10, p504s, C-KIT, and CK7
13	82	m	Single	One-sided	2.6	6.19	R	Positive for CD10, p504s, and C-KIT
14	74	m	Single	One-sided	2.6	6.78	R	Positive for CD10, C-KIT, p504s, and CK7
15	83	m	Single	One-sided	4	1.99	L	Positive for MNF116, CK7, CD10, C-KIT, and p504s
16	75	f	Single	One-sided	1.6	10.38	R	Positive for E-cadherin
17, 1	72	m	Multiple	Bilateral		0.32	L	None performed
17, 2	72	m	Multiple	Bilateral		0.46	R	None performed
18	92	m	Single	One-sided	4.4	2.41	R	None performed
19	29	f	Single	One-sided	3	0.00	L	Stains used

## Data Availability

The data used to support the findings of this study are included within the article.
